# Cloning and bioactivity analysis of a CXC ligand in black seabream *Acanthopagrus schlegeli*: the evolutionary clues of ELR^+^CXC chemokines

**DOI:** 10.1186/1471-2172-9-66

**Published:** 2008-11-07

**Authors:** Cai Zhonghua, Gao Chunpin, Zhang Yong, Xing Kezhi, Zhang Yaou

**Affiliations:** 1 Life Science Division, Graduate School at Shenzhen, Tsinghua University, Shenzhen, PR China; 2 Tianjin Key Lab of Aqua-ecology and Aquaculture, Tianjin Agricultural University, Tianjin, PR China

## Abstract

**Background:**

The ELR^+^CXC chemokines are multifunctional mediators that are mainly responsible for the recruitment of leucocytes to sites of inflammation and infection. Because of their high sequence identity with mammalian IL-8, fish IL-8-like CXCs have been named as piscine 'IL-8' and included in the ELR^+ ^subgroup, even though there is no reliable functional or evolutionary evidence to support this classification.

**Results:**

In this investigation, a homologue of piscine 'IL-8' from black seabream (*Acanthopagrus schlegeli)*, called BS CXC, has been cloned and analyzed. The results revealed that BS CXC has a high gene similarity and tertiary structure similarity with piscine and mammalian CXC chemokines, both ELR^-^CXC and ELR^+^CXC, although it has a lower identity with ELR^-^CXC, compared with ELR^+^CXC chemokines. Like other piscine IL-8, BS CXC has only an incomplete ELR motif, which is essential for the mammalian ELR^+^CXC ability to attract granulocytes. Bioactivity assay demonstrated that the BS rCXC produced in *E. coli *significantly stimulated migration of fish neutrophils and macrophages, but had no effect on rat neutrophils and macrophages, whereas hrIL-8 induced strong chemotaxis of fish neutrophils but did not affect fish macrophages. BS CXC seems show some structural and functional properties of the intermediate between ELR^-^CXC and ELR^+^CXC.

**Conclusion:**

As an incomplete ELR^+^CXC chemokine from a modern fish, BS CXC provides some clues on the evolution from ancient ELR^-^CXC to ELR^+^CXC by retaining some properties of the intermediate stage in evolution, and it may be more appropriate to call this molecule 'piscine CXC with an incomplete ELR', instead of terming it fish 'IL-8'.

## Background

Chemokines are a group of small peptide chemotactic cytokines, which are multifunctional mediators that can trigger inflammatory cell chemotaxis toward a site of infection and injury by binding to a G-protein-coupled cell surface receptor [1,2]. Chemokines have pleiotropic effects in regulating immunity and angiogenesis, and stem cell trafficking appears to play a central role in linking innate and acquired immune regulation [3,4]. Functionally, chemokines fall into two main categories; one is homeostatic and generally involved in lymphocyte trafficking, immune surveillance and localization of lymphocytes; the other category is only produced by cells during inflammation to prompt the migration of leukocytes to an injured or infected site and also activates cells to raise an immune response and commence the wound healing process. [5]. Based on structural properties and primary amino acid sequence, chemokines are divided into four groups, including the CXC, CC, C and CXXXC subfamily, according to the position of the first two cysteines [6,7]. Nearly 50 different CXC and CC chemokines have been identified and well studied in human and mammalian cells by biochemical purification or cDNA-deduced amino acid sequencing [5,8].

CXC chemokines can be further subdivided into those that contain a short sequence of Glu-Leu-Arg (the ELR motif) and those that do not, the ELR^+ ^subgroup and the ELR^- ^subgroup [9,10]. The ELR^+^CXC specifically recruits polymorphonuclear leucocytes (PMN) into inflamed tissues and promotes angiogenesis by specifically binding to CXCR1 and/or CXCR2 [10], whilst ELR^-^CXC specifically attracts lymphocytes and monocytes, with poor chemotactic ability for neutrophils, and inhibits angiogenesis [3,5,9].

In recent years, the progress of nonmammalian chemokine research has been rapid since the first 'IL-8' homologue was cloned in lamprey [11]. Although many gene products are identified by the molecular cloning approach [12-19], little information is available on their biological effects. Because of their high sequence identity with mammalian IL-8, fish IL-8-like CXCs have been named as piscine 'IL-8' and included in the ELR^+ ^subgroup, even though there is no reliable functional or evolutionary evidence to support this classification. In this paper, we cloned a homologue of piscine 'IL-8' from black seabream (*Acanthopagrus schlegeli*), called BS CXC, and analyzed its sequence and bioactivities. Finally, we used the BS CXC as a model to evaluate the position of piscine 'IL-8' in the evolutionary development of chemokines. This investigation thus provided some insights into the evolution of chemokines.

## Results

### Cloning and sequence analysis of the BS IL-8-like gene

**Figure 1:**
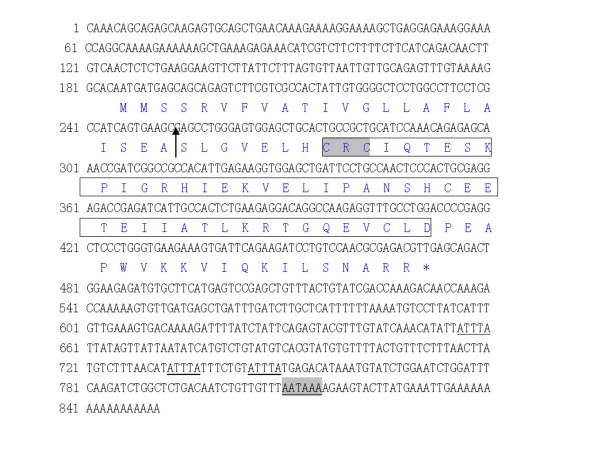
**Sequence of BS CXC**. The nucleotide sequences of BS CXC are represented by black letters and the deduced amino acid sequences by blue letters. The arrow indicates the signal peptide cut site; the CXC subfamily signature is in the box and the CXC motif is in the highlighted box; the RNA instability motif is underlined and the polyadenylation signal sequence is highlighted and underlined; the asterisk shows the stop code. Accession numbers of BS CXC is DQ000611.

A 175 bp sequence was cloned by PCR using a pair of degenerated primers designed from the conserved region of the mammalian and piscine CXC sequence and the sequence showing relatively higher identity and similarity with the known mammalian and piscine CXC ligands (E < e^-10^). Subsequently, two specific primers of IL-8F85 and IL-8R94 were designed to get the 3' and 5' ends of BS CXC cDNA, respectively. The products of 360 bp and 492 bp were amplified by RACE. Compiling the three overlapped sequences gave a consensus sequence of 851 bp, which represents the full coding sequence of BS CXC cDNA (Fig. 1), then, we designed the other pair of primers, gF and gR which covers the full ORF coding sequence region of the candidate BS CXC to get the full length transcript of BS CXC with RT-PCR. The product of RT-PCR was sequenced and the full length transcript of BS CXC was confirmed.

The BS CXC sequence contains an 83 bp 5' untranslated region (UTR) and a 285 bp open reading frame (ORF) encoding a propeptide of 95 amino acids, including a signal peptide of 22 amino acids. The deduced peptide has a putative molecular weight (MW) of 10.5 kDa, a theoretical isoelectric point of 7.86, and a 380 bp 3' UTR, which contains three RNA unstable motifs (ATTTA) and a typical polyadenylation signal 18 bp upstream of the poly A tails. The sequence was deposited in Genbank with the accession No. of DQ000611.

**Figure 2:**
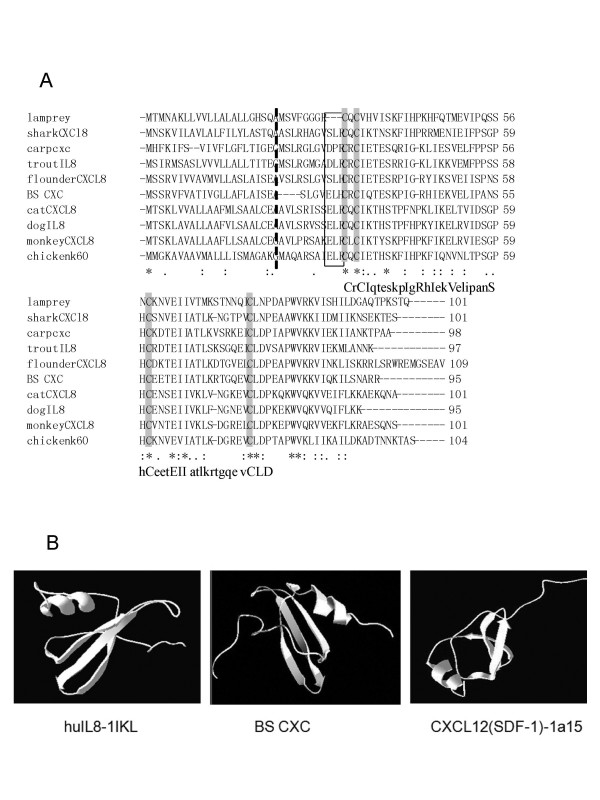
**Amino acid sequence alignment and Comparison of tertiary structure of BS CXC**. A. Amino acid sequence alignment of BS CXC with other known CXCL8. The alignment was performed with the Clustal W program. Identical and similar sites are shown with asterisks (*) and dots (. or :), respectively; the broken line indicates the signal peptide cut site. The conserved cysteines are highlighted, and the ELR motif associated with attracting neutrophils in mammals is in the box. B. Comparison of tertiary structure among the human IL-8 model pdb 1Ikl (left), BS CXC (middle) and the CXCL12 (SDF-1) model pdb 1a15. The BS CXC models were predicted by the SWISS-MODEL and Swiss-PdbView software.

The putative peptide of BS CXC contains a typical structure of the chemokine CXC ligand, which is formed by two cysteines separated by Arg. The four cysteines that should be involved in forming a disulphide bridge are highly conserved, and the CXC chemokine family signature of C-x-C- [LIVM]-x(5,6)- [LIVMFY]-x(1,2)- [RKSEQ] -x- [LIVM]-x(2)- [LIVM]-x(5)- [STAG]-x(2)-C-x(3)- [EQ]- [LIVM](2)-x(9,10)-C-L- [DN] [20] was identified in BS CXC (Fig. 2A). The multiple alignments of fish and other animal CXC peptides also indicate that most of the conserved amino acids are found in the mature peptide region, especially in the signature, forming secondary structure regions. The tertiary structure predicted by the SWISS-MODEL shows that the BS CXC had a similar structure to piscine and mammalian CXC chemokines, both ELR^+^CXC and ELR^-^CXC, which contains three β strands in the N terminal and a α helix in the C terminal, and this is in common with the results of human CXC X-rays [21-24] (Fig. 2B).

Structural analysis uncovered significant differences in receptor binding regions; like other piscine 'IL-8', the BS CXC putative peptide lacks a complete ELR motif, which is important for its chemotaxis of the neutrophils, because the motif is the essential domain in the ELR^+^CXC ligand binding to the receptor [25]. BS CXC and all the other fish 'IL-8' only have an incomplete ELR motif; for example, the Arg was substituted by His to be the ELH in BS CXC. For the DLR motif in rainbow trout, the Glu was replaced by Asp [12], for the SLH motif in flounder [17], the Glu and Arg were substituted by Ser and His, respectively. The other piscine CXCs also have an incomplete ELR motif (Fig. 2A).

Alignment of amino acid sequences of the BS CXC with the other CXC chemokines indicates that the BS CXC has a higher similarity with all the CXC chemokines (36–72%), both ELR^+^CXC(72-42%) and ELR^-^CXC(49-36%), and has higher identity with ELR^+^CXC (60.5-25.9) but a lower identity with ELR^-^CXC(28.1-16.1%) (Fig. 2A). The BS CXC has the highest identity with bony fish IL-8 (60.5-51%), then the cartilaginous banded dogfish *Triakis scyllia *(43.1%) and mammalian ELR^+^CXC (25.9–40%). But the Agnatha (jawless fishes) lamprey only has a lower identity (33.6-18.6%) with all the CXC chemokines [17].

### Phylogenetic analysis

**Figure 3:**
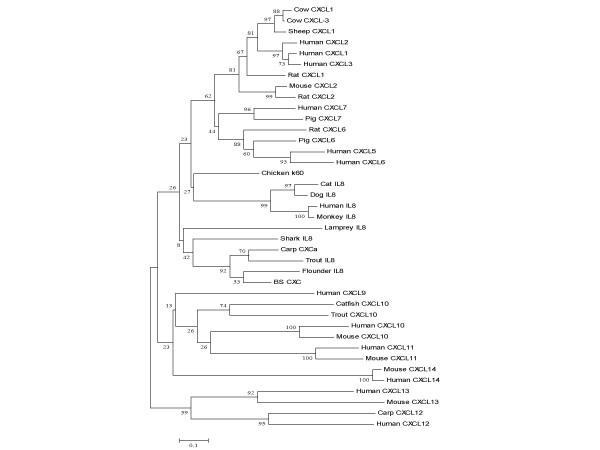
**Phylogenetic analysis**. The sequences were aligned by the CLUSTAL W program and the phylogenetic tree was constructed by neighbor-joining methods using MEGA version 2.1. The phylogenetic tree shows the relationship among the full-length amino acid sequences of the BS CXC mature peptide with other representative CXC sequences, Numbers at branch nodes represent the confidence level of 1000 bootstrap replications. The used sequences are represented as follows: human CXCL1 AAP36752; human CXCL3 NP_002081; human CXCL4_p02776; human CXCL5 NP_002985; human CXCL6 NP_002984; human CXCL7 NP_002695; human IL-8 NP_000575; human CXCL11 O14625; human CXCL12_P48061; human CXCL13_O43927; human CXCL14 O95715; chicken K60_CAA75212; *Triakis scyllium *IL-8 BAB79448; trout IL-8 CAC33585; lamprey IL-8 CAA13114; flounder IL-8 AAL05442; carp CXCa CAD13189; BS CXC AAY18807; cow CXCL1 NP_783631; cow CXCL3 NP_776724; sheep CXCL1 AAB93930; mouse CXCl 2 NP_033166; rat CXCL1 NP_110472; Rat CXCL2 NP_446099; pig CXCL7 AAB28904; carp CXCL12 CAD59916; catfish CXCL10 AAQ01585; trout CXCL10 CAD01141; rat CXCL3 BAA02009; rat CXCL6 NP_071550; dog IL-8 JN0841; monkey IL-8 AAA86705; mouse CXCL10 P17515; mouse CXCL11 Q9JHH5; mouse CXCL13 O55038; mouse CXCL14 Q9WUQ6).

A phylogenetic tree was constructed based on the mature peptide of BS CXC and part of the known CXC ligands by using the neighbour-joining algorithm (Fig. 3). The ELR^+^CXC of mammalian CXC and incomplete ELR^+^CXC of piscine CXC, which are orthologous genes that represent independent lineages and have diverged from ELR^-^CXC, share sister-taxon relationships on the evolutionary tree. All the piscine incomplete ELR^+^CXC chemokines formed one clade. In this clade, lamprey CXC of the *Agnatha *(jawless fishes) is in an independent branch that evolved first from ancestral CXC and later from cartilaginous fish CXC and other bony fish CXC. In the mammalian ELR^+^CXC clade, the orthologous genes of IL-8 and chicken K60 ligands share parallel evolutionary relationships, with the orthologous genes of CXC 5-7 and CXC 1–3 forming sister branches. Piscine incomplete ELR^+^CXC chemokines originated almost contemporaneously through a series of gene duplication events. There is no solid evidence to confirm that fish incomplete CXC were clearly orthologous with mammalian IL-8. The results indicate that the ELR^+^CXC chemokine ligands may have evolved from ancestral ELR^-^CXC and diverged further during evolution to perform a special function. Fish incomplete ELR^+^CXC retain some properties of the intermediate stage during the evolutionary development of highly specific CXC chemokines.

### Expression of natural and recombinant BS CXC

**Figure 4:**
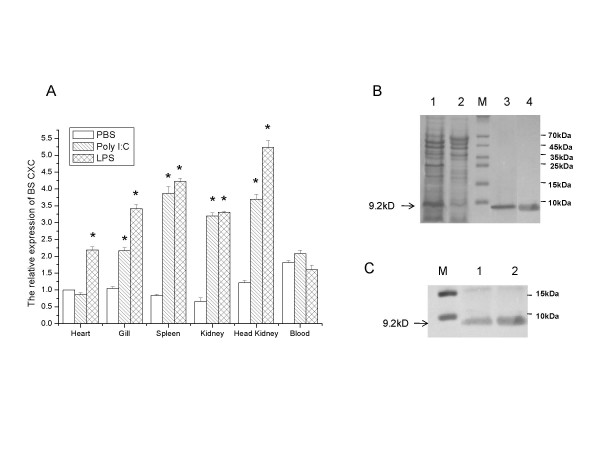
**Expression analysis of the BS CXC**. (A) The expression of BSCXC mRNA relative to b-actin analyzed by real-time PCR in Black Seabream after PolyI:C and LPS challenge. The values are shown as means ± S.E., n = 3. Significant differences between challenged group and control group are indicated by an asterisk (P < 0.01). (B) The plasmid BS CXC-pQE30 was transformed into *E. coli *M15 cells and induced by ITPG. Bacterial lysates (lane 1) or purified BS rCXC (lanes 3 and 4) were separated on SDS-PAGE and stained by Coomassie brilliant blue R250. Lysate from uninduced cells was used as a control (lane 2). (C) The identity of the BS rCXC was confirmed by Western blotting using the antibody of 6His. Lanes 1 and 2 showed BS rCXC.

Fluorescent real-time quantitative PCR was employed to measure the expression of the BS CXC transcript in the tissues of normal and challenged black seabream (*Acanthopagrus schlegeli*). The results showed basal expression of BS CXC in various organs, including the heart, gill, spleen, kidney and HK, and the basal expression was higher in the blood than in other tissues (Fig. 4A). Figure 4A also demonstrated that poly(I:C) and LPS can significantly up-regulate BS CXC transcript expression in various organs. Twenty-four hours after stimulation with poly(I:C) and LPS, more than 4-fold up-regulation of BS CXC transcript expression was detected in the HK and spleen tissue and more than 2-fold transcript expression was detected in the kidney, gill and other tissues. LPS seems to have a stronger effect than poly(I:C) in the up-regulation of BS CXC expression. The results demonstrate that BS CXC expression can be induced by poly(I:C) or LPS, as in the case of other chemokines and cytokines [14].

To get purified recombinant protein for the functional assay of BS CXC, we generated a BS CXC expression construct through inserting a BS CXC mature peptide coding sequence into vector pQE30 (Qiagen). The construct was transformed into *E. coli *M15 cells and the cells were induced to produce the recombinant BS CXC containing a 6 His tag at the N-terminus for purification. Figure 4B shows that the recombinant protein significantly increased before (lane 2) and after induction (lane 1). The molecular size of the recombinant protein is about 9.2 kD, matching well with the predicted molecular mass weight. Lanes 3 and 4 show the purified protein. The BS rCXC protein was confirmed by Western blot analysis using an mAb against the histidine tag, in which a single band was detected (Fig. 4C).

### Chemotactic response of leucocytes towards the BS CXC gene

**Figure 5:**
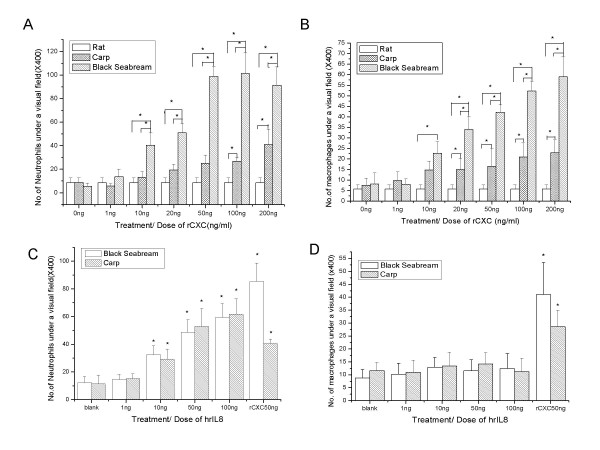
**Migratory assay of leukocytes from different animals towards BS rCXC or human rIL-8**. The migration of neutrophilic granulocytes (A and C) or macrophages (B and D) from black seabream, carp and rat induced by different concentrations of BS rCXC (A and B) or human IL-8 (C and D) was assayed using Transwell.*: p < 0.05.

According to the sequence analysis, BS CXC is a putative CXC chemokine with an incomplete ELR, so the chemokine might have the functional characters of both ELR^+^CXC and ELR^-^CXC. A migratory assay of leukocytes was performed to test our hypothesis. The neutrophils were collected from peripheral blood and macrophages were isolated from the lungs of rats and the head kidneys of black seabream and common carp. Dose-dependent chemotactic responses induced by BS CXC were observed in the migratory assays of BS neutrophils and macrophages (Fig. 5A and 5B). The chemotactic activity appeared at low doses of BS rCXC (10 ng/ml), then became stronger with increasing doses of BS rCXC, and peaked at doses of 100 ng/ml for induction of neutrophils and 200 ng/ml for macrophages. The BS rCXC showed stronger chemotactic activity to BS neutrophils than to BS macrophages. This chemokine can also induce migration of carp neutrophils and macrophages, though its chemotactic activity to carp leucocytes was significantly lower than that to those of black seabream. However, neutrophils from higher vertebrates, such as the rat, did not show any chemotactic responses to BS rCXC. Our results suggest that BS rCXC can induce chemotactic responses in both neutrophils and macrophages of fish, but not mammalian, cells.

Piscine CXC could not induce mammalian cells, but we did not know whether mammalian CXC could affect fish cells or not. Human recombinant IL-8 (hrIL-8) was used to do a migratory assay of fish neutrophils. As shown in Fig. 5C and 5D, hrIL-8 induced remarkable chemotaxis of fish neutrophils but did not affect fish macrophages both in Black Seabream and Common Carp.

## Discussion

The first reported fish chemokine gene from a jawless fish lamprey belonged to the CXC family; the gene encodes an IL-8-like peptide and was called lamprey 'IL-8'. Following these discoveries, similar molecules were identified in different fishes, including shark, trout, flounder and carp [26]. Because their sequences have a high identity with lamprey IL-8, these piscine CXCs were called fish 'IL-8 '; however, the name does not seem suitable, at least in the case of BS CXC.

CXC chemokine ligands are grouped into two subgroups, ELR^+^CXC and ELR^-^CXC in different species. Mammalian IL-8 belongs to the ELR^+^CXC subgroup of CXC chemokines containing the ELR motif (ELR^+^). This group includes CXCL1 to 8, except CXCL4, and these chemokines have implications in the specificity for granulocytes. In contrast, the ELR^- ^subgroup includes CXCL4 and CXCL9 to 14, and these chemokines attract lymphocytes and monocytes, with poor chemotactic ability for granulocytes [5,14,27,28]. Fish 'IL-8' are grouped into the ELR^+ ^subgroup but the classification seems unsuitable [14,29], not only because of their incomplete ELR motif, but also because of their functional differences compared with mammalian IL-8.

In this study, we constructed a phylogenetic tree using the neighbour-joining algorithm (Fig. 3) and a similar topology by the maximum-parsimony algorithm (data not shown). Based on our results from phylogenetic analysis and those obtained by others, CXCL12 and CXCL13 are, in phylogenetic terms, modern representatives of the ancestral ELR^-^CXC chemokine [7,14], and mammalian ELR^+^CXC and piscine incomplete ELR^+^CXC are derived from ancestral ELR^-^CXC. Piscine incomplete ELR^+^CXC represents an early evolutionary stage of ELR^+^CXC. Among the fishes, lamprey 'IL-8' (jawless fishes), which occupies an independent branch, may be the first that evolved from the ELR^-^CXC ancestor, followed by shark 'IL-8' (cartilaginous fish), and finally leading to common carp 'IL-8', rainbow trout 'IL-8', flounder 'IL-8' and BS CXC, etc (bony fishes). Similar to the CXC protein molecules, there is also an evolutionary trend for the fish ELR-like motif, from GGR (jawless fishes) to SLR/SLQ (cartilaginous fish), then to SLH/DLR/ELH (bony fishes), and finally to ELR (birds and mammals). The entire process has probably occurred through multi-time mutations of amino acids that have led to the formation of an ELR motif–a special binding site for the CXC receptor in mammals.

In mammalian species, all CXC chemokines have a similar tertiary structure which contains three β strands in the N terminal and an α helix in the C terminal, and all the functional CXC chemokines act by binding to G-protein-coupled cell surface receptors via a seven-transmembrane-domain, and signaling through a heterotrimeric GTP-binding protein [9,30]. Therefore, it is possible that all the CXC chemokines evolved from an ancestral gene through multiple duplication (7). The low identity among CXC genes maybe result in conservative substitutions of amino acids to suitable function-specific diversification in the early evolutionary stages (14).

The differences in molecular structures hint at differences in functions between mammalian IL-8 and fish 'IL-8'. Before this investigation, almost no functional investigations about fish 'IL-8' had been performed, so it was unclear whether fish 'IL-8' and mammalian IL-8 have the same function or not. In this study, we used BS CXC as a model of fish 'IL-8' to investigate the chemotactic functions of fish 'IL-8' and found that BS CXC developed a chemotactic ability for granulocytes, but still kept the chemotactic property of inducing macrophages and neutrophils, compared with its ancestors of the ELR^-^CXC chemokine. Unlike fish 'IL-8', the mammalian IL-8 evolved a more specialized chemotactic function, inducing only granulocytes, but lost its attraction for macrophages. It is generally acknowledged that the ELR motif is an essential structure for the chemotaxis of granulocytes [25], however, our investigation showed that the incomplete ELR motif of fish 'IL-8', like the ELH of BS CXC, already has chemotactic ability for fish granulocytes, but mammalian IL-8 has a stronger chemotactic ability for granulocytes, not only inducing mammalian granulocytes, but also affecting fish granulocytes. Our results are supported by an experiment on an ELR mutation of human IL-8. When the ELR motif of human IL-8 molecule was mutated to an incomplete ELR motif, such as ALR, EAR or ELA, the mutated human IL-8 retained its neutrophil attracting ability, but also showed a 100 to 1000 fold reduced affinity to bind with neutrophil receptors [25]. These data suggests that the ELR motif is essential for mammalian cells, but in fish incomplete ELR CXC is also functional for the chemotaxis of granulocytes.

Taken together, although fish 'IL-8' with an incomplete ELR motif already has granulocyte-attracting ability similar to mammalian IL-8, it still retains a strong chemotactic ability for monocytes, similar to its ancestor, the ELR^-^CXC chemokine. It seems that fish incomplete ELR^+^CXC still retain some properties of the intermediate stage during the evolution from ancient ELR^-^CXC to ELR^+^CXC chemokines. Therefore, several papers have noted that fish IL-8 is an inappropriate name for piscine chemokines (14, 28), and it may be more appropriate to call this molecule 'piscine CXC with an incomplete ELR', instead of terming it fish 'IL-8'.

## Conclusion

A homologue of piscine 'IL-8' from black seabream, called BS CXC, has been cloned and analyzed. The results revealed that BS CXC has a high gene similarity and tertiary structure similarity with piscine and mammalian CXC chemokines, both ELR^+^CXC and ELR^-^CXC, although it has a lower identity with ELR^-^CXC, compared with ELR^+^CXC chemokines. Like other piscine IL-8, BS CXC has only an incomplete ELR motif, which is essential for the mammalian ELR^+^CXC ability to attract granulocytes. Bioactivity assay demonstrated that the BS rCXC significantly stimulated migration of fish neutrophils and macrophages. As an incomplete ELR^+^CXC chemokine from a modern fish, BS CXC provides some clues on the evolution from ancient ELR^-^CXC to ELR^+^CXC by retaining some properties of the intermediate stage in evolution.

## Methods

### Fish

Black seabream (*Acanthopagrus schlegeli*), weighing about 100 g, were collected from a local fish farm in Shenzhen, Guangdong province, and acclimated for 10 days in a 200 L fiberglass tank containing clean seawater at 25 ± 3°C. Each day 25% of the water was replaced by fresh seawater. Fish were fed commercial food at a daily rate of 1.5% of their estimated body weight. All the fishes were handled according to the regulations of "U.S. Government Principles for the Utilization and Care of Vertebrate Animals Used in Testing, Research, and Training".

### RNA isolation and cDNA synthesis

Healthy black seabream were challenged with an attenuated marine fish bacterial pathogen of *Vibrio anguillarum *(about 2 × 10^8 ^cells per fish). Then 24 hours after stimulation, the head kidney HK was isolated and homogenized for RNA isolation. Total RNA was extracted by Trizol reagent (Invitrogen, Japan) following the user's manual. The cDNA was synthesized from total RNA by MMLV reverse transcriptase (Promega, USA) with oligo dT or adapter dT primer.

### Cloning and sequencing of the CXC homologue

PCR (polymerase chain reaction) was performed using the cDNA template prepared above and a pair of degenerated primers dCXC-F/dCXC-R (Additional file 1), which were designed based on the conserved regions of the known CXC sequences, to amplify the black seabream CXC (BS CXC) chemokine homologue. The PCR products were ligated into pMD18-T vector (TaKaRa, Dalian) and transformed into XL1-blue *E. coli *competent cells. Plasmid DNA was isolated using a GenElute plasmid miniprep Kit (Sigma). The insert in the plasmid DNA was sequenced using the ABI377a Automated Sequencer (Applied Biosystems).

Based on the partial sequence of BS CXC, the 5' ends and 3' ends were obtained by rapid amplification of cDNA ends (RACE) approaches [31], using gene specific primers and adapter primers (F85/adapter primer; oligo dG/R94) (Additional file 1). The PCR products were cloned to pMD-18T vector and sequenced.

Based on the candidate BS CXC sequence, a pair of primer gF(5' -ATG ATG AGC AGC AGA GTC- 3')/gR (5'-CAG ATT GTC AGA GCC AGA-3'), which was located in 5'UTR and 3'UTR of the BS CXC sequence, respectively, was designed. RT-PCR was carried out with these primers to get the full length transcript of BS CXC.

### Sequence analysis

The nucleotide sequences were analyzed by the BLAST [32] program to determine gene identities, and multiple sequence alignment was generated using the CLUSTAL W [33] program. The signal peptide prediction was performed by the SignalP program [34] and the protein family signature was identified by the InterPro program [35]. The tertiary structure was predicted by the SWISS-MODEL software [36] and visualized by the Swiss-PdbView software [37]. The phylogenetic tree was constructed based on the full-length amino acid sequences of part of the known mammalian and piscine CXC ligands, using a neighbor-joining algorithm within MEGA version 3.1 [38].

### Expression studies

Expression of the BS CXC transcript was studied by fluorescence real-time RT-PCR. Healthy black seabream were challenged with LPS (Sigma) or poly(I:C) (Sigma) at a dose of 3 mg/kg body weight, and the PBS-challenged fish were used as the negative control. Twenty-four hours after stimulation, total RNA was extracted from different organs, including the gill, heart, head kidney (HK), kidney, spleen and blood. Single-strand cDNA was synthesized with MMLV reverse transcriptase (Promega) and the oligo dT primer (Promega) using DNase I (Promega)-treated total RNA as the template. The gene-specific primer F85 and GR primers (Additional file 1) were used to amplify the targets, and the fragment of the β-actin gene that was amplified by β-actin-F/β-actin-R was used as the internal control to verify quantitative real-time PCR and successful transcription. The fluorescent real-time PCR assay was carried out in an ABI PRISM 7300 Sequence Detection System (Applied Biosystems). The amplifications were performed in triplicates in 20-μL reaction volume that contained 10 μL 2× SYBR Green Master Mix (Applied Biosystems), 1 μL (each) forward and reverse primers (10 μmol L^-1^), 3 μL cDNA and 5 μL DEPC-treated water. The thermal program for real-time PCR was 95°C for 5 min followed by 35 cycles at 95°C for 30 s and 61°C for 1 min. Dissociation curve analysis of amplification products was performed at the end of each PCR reaction to confirm that only one PCR product was amplified and detected. After the PCR program, fluorescent real-time PCR data from three replicate samples were analyzed with 7300 System SDS Software v.1.4.0 (Applied Biosystems, USA). The comparative CT method was used to analyze the BS CXC expression level. All analyses were based on the CT values of the PCR products. The CT was defined as the PCR cycle at which the fluorescence signal crossed a threshold line that was placed in the exponential phase of the amplification curve. The CT for target amplification of BS CXC and that for the internal control β-actin were determined for each sample. The difference between the CT values of the target and internal control, termed dCT, was calculated to normalize the differences in the amount of total nucleic acid added to each reaction and the efficiency of the RT-PCR. One of the samples was chosen as the reference sample and was called the calibrator. The dCT for each sample was subtracted from the dCT of the calibrator, and the difference gave the ddCT value. The BS CXC expression level could be calculated by 2^-ddCT^; the value represented an n-fold difference relative to the calibrator. The data obtained from real-time PCR were subjected to the Student's *t*-test to determine differences in the mean values between the control and treated groups. P values less than 0.01 were regarded as significant.

### Construction and expression of recombinant BS CXC

The cDNA encoding sequences of the deduced mature peptide were amplified by PCR using the gene specific primers rCXC F/rCXC R, which contained restriction enzyme sites for *BamHI *or *Hind III*, respectively (Additional file 1). The digested PCR products by *Bam HI *and *Hind III *were ligated into expression vector pQE30 (Qiagen) containing a 6 His tag at the N-terminus for purification purposes and cloned into *E. coli *competent cells M15. The positive clones were screened by PCR with primers pQE30 F/rCXC R and the plasmids of positive clones were purified and sequenced using pQE30-R primer (Additional file 1). The resultant proteins were named BS rCXC.

The positive colony was cultured and induced by IPTG, and then the purified recombinant protein was generated using a Ni-NTA agarose kit (Qiagen) under denaturing conditions, following the user manual. The protein was dialyzed in refolding buffer (50 mM PBS containing 1 mM GSH, 0.1 mM GSSH, 1 mg/ml leupeptin and 1 mg/ml pepstatin). Expression and purified protein were checked by 4–18% SDS PAGE gel. Western blot analysis was performed to confirm the identity of the BS rCXC; the monoclonal antibody of 6His (Upstate, USA) was used as the primary antibody for the detection of the Western blot.

### Preparation of primary cultured leukocytes

Head kidney (HK) macrophages and blood neutrophils were isolated according to the method of Solem J (1995) [39] and refined. Briefly, the head kidney of black seabream or common carp was surgically removed, then passed through a 100 um nylon mesh with ice cold L-15 containing 10 U/mL heparin, 100 ug/ml penicillin and streptomycin (P/S). After washing several times, the cells were resuspended in ice cold L-15. The macrophages and blood neutrophils were purified by a discontinuous Percoll density gradient, collecting more than 80% of the neutrophils and over 90% of the macrophages in the purified cells.

Rat blood neutrophils were isolated like fish blood neutrophils isolation, and Rat macrophages were isolated from rat lungs by the perfusion method. Briefly, the lungs and trachea were picked out carefully, HBSS was added from the trachea to wash and perfuse the lung macrophages. The perfusion liquid containing macrophages was sedimented at 400 × g for 10 min at 4°C. The pellet was washed and resuspended with RPMI 1640 medium (containing 15 mmol/L HEPES, 0.05 U/ml insulin, 100 μg/ml P/S) supplemented with 5% fetal calf serum. The viable cells were determined by 0.5% trypan blue exclusion. All the rats were handled according to the principles of "Public Health Service Policy on Humane Care and Use of Laboratory Animals".

### Migration assays

The migration of the macrophages and the blood neutrophil cells induced by different concentrations of BS rCXC or hrIl8 was investigated using Transwell[40] permeable supports (Corning, USA), following the user manual. Briefly, after equilibrium the polycarbonate membrane with a 0.5 um diameter pore was used in the Transwell inserts with a culture medium overnight at 25°C or 37°C, respectively, the BS rCXC or hrIL8 dissolved in fresh L15 culture medium containing 5% fetal calf serum, 10 U/mL heparin and 100 ug/ml P/S was added to the plates, then 0.5 ml 1 × 10^6 ^cell/ml of the neutrophils or macrophages in fresh medium were added to the Transwell inserts; each dose had three replicates. After 2 h incubation at 25°C or 37°C in a humid chamber, the polycarbonate membranes were removed from the insert, and the upper layer cells of the membrane were rinsed with HBSS to remove the cell residues. Then the membranes were fixed in methanol and stained by Giemsa stain and the migration cells passing through the polycarbonate membranes were counted in twenty randomly selected fields under the microscope.

### Statistical analysis

The means and standard errors were calculated from the replicates. The mean values were compared using the Origin 6.0 program (Origin Lab Corporation, USA). A one-way ANOVA test was performed when significant differences occurred at the 5% level. Differences were considered significant at p-values of < 0.05.

## Authors' contributions

CZ Conceived and designed the study and drafted the manuscript. GC, ZY and X K Performed the experiments and analyzed the data. ZYO participated in design of the study, helped to draft the manuscript and revised the manuscript.

## Supplementary Material

Additional file 1 **Table for cxc primer** Click here for file

## References

[B1] Kupper RW, Dewald B, Jakobs KH, Baggiolini M, Gierschik P (1992). G-protein activation by interleukin 8 and related cytokines in human neutrophil plasma membranes. Biochem J.

[B2] Campbell JJ, Hedrick J, Zlotnik A, Siani MA, Thompson DA, Butcher EC (1998). Chemokines and the arrest of lymphocytes rolling under flow conditions. Science.

[B3] Strieter RM, Burdick MD, Mestas J, Gomperts B, Keane MP, Belperio JA (2006). Cancer CXC chemokine networks and tumour angiogenesis. Eur J Cancer.

[B4] Yoshie O, Imai T, Nomiyama H (2001). Chemokines in immunity. Adv Immunol.

[B5] Laing KJ, Secombes CJ (2004). Chemokines. Dev Comp Immunol.

[B6] Zlotnik A, Yoshie O (2000). Chemokines: a new classification system and their role in immunity. Immunity.

[B7] Modi WS, Yoshimura T (1999). Isolation of novel GRO genes and a phylogenetic analysis of the CXC chemokine subfamily in mammals. Mol Biol Evol.

[B8] Murphy PM, Baggiolini M, Charo IF, Hebert CA, Horuk R, Matsushima K, Miller LH, Oppenheim JJ, Power CA (2000). International union of pharmacology. XXII. Nomenclature for chemokine receptors. Pharmacol Rev.

[B9] Baggiolini M, Dewald B, Moser B (1997). Human chemokines: an update. Annu Rev Immunol.

[B10] Bizzarri C, Beccari AR, Bertini R, Cavicchia MR, Giorgini S, Allegretti M (2006). ELR+ CXC chemokines and their receptors (CXC chemokine receptor 1 and CXC chemokine receptor 2) as new therapeutic targets. Pharmacol Ther.

[B11] Najakshin AM, Mechetina LV, Alabyev BY, Taranin AV (1999). Identification of an IL-8 homolog in lamprey (Lampetra fluviatilis): early evolutionary divergence of chemokines. Eur J Immunol.

[B12] Laing KJ, Bols N, Secombes CJ (2002). A CXC chemokine sequence isolated from the rainbow trout Oncorhynchus mykiss resembles the closely related interferon-gamma-inducible chemokines CXCL9, CXCL10 and CXCL11. Eur Cytokine Netw.

[B13] Fujiki K, Shin DH, Nakao M, Yano T (1999). Molecular cloning of carp (Cyprinus carpio) CC chemokine, CXC chemokine receptors, allograft inflammatory factor-1, and natural killer cell enhancing factor by use of suppression subtractive hybridization. Immunogenetics.

[B14] Huising MO, Stet RJ, Kruiswijk CP, Savelkoul HF, Lidy Verburg-van Kemenade BM (2003). Molecular evolution of CXC chemokines: extant CXC chemokines originate from the CNS. Trends Immunol.

[B15] Inoue Y, Endo M, Haruta C, Taniuchi T, Moritomo T, Nakanishi T (2003). Molecular cloning and sequencing of the silver chimaera (Chimaera phantasma) interleukin-8 cDNA. Fish Shellfish Immunol.

[B16] Inoue Y, Haruta C, Usui K, Moritomo T, Nakanishi T (2003). Molecular cloning and sequencing of the banded dogfish (Triakis scyllia) interleukin-8 cDNA. Fish Shellfish Immunol.

[B17] Lee EY, Park HH, Kim YT, Choi TJ (2001). Cloning and sequence analysis of the interleukin-8 gene from flounder (Paralichthys olivaceous). Gene.

[B18] Noubissi FK, Elcheva I, Bhatia N, Shakoori A, Ougolkov A, Liu J, Minamoto T, Ross J, Fuchs SY, Spiegelman VS (2006). CRD-BP mediates stabilization of betaTrCP1 and c-myc mRNA in response to beta-catenin signalling. Nature.

[B19] Baoprasertkul P, Peatman E, Chen L, He C, Kucuktas H, Li P, Simmons M, Liu Z (2004). Sequence analysis and expression of a CXC chemokine in resistant and susceptible catfish after infection of Edwardsiella ictaluri. Dev Comp Immunol.

[B20] Oppenheim JJ, Zachariae CO, Mukaida N, Matsushima K (1991). Properties of the novel proinflammatory supergene "intercrine" cytokine family. Annu Rev Immunol.

[B21] Baldwin ET, Franklin KA, Appella E, Yamada M, Matsushima K, Wlodawer A, Weber IT (1990). Crystallization of human interleukin-8. A protein chemotactic for neutrophils and T-lymphocytes. J Biol Chem.

[B22] Rajarathnam K, Clark-Lewis I, Sykes BD (1995). 1H NMR solution structure of an active monomeric interleukin-8. Biochemistry.

[B23] Montserret R, McLeish MJ, Bockmann A, Geourjon C, Penin F (2000). Involvement of electrostatic interactions in the mechanism of peptide folding induced by sodium dodecyl sulfate binding. Biochemistry.

[B24] Dealwis C, Fernandez EJ, Thompson DA, Simon RJ, Siani MA, Lolis E (1998). Crystal structure of chemically synthesized [N33A] stromal cell-derived factor 1alpha, a potent ligand for the HIV-1 "fusin" coreceptor. Proc Natl Acad Sci USA.

[B25] Hebert CA, Vitangcol RV, Baker JB (1991). Scanning mutagenesis of interleukin-8 identifies a cluster of residues required for receptor binding. J Biol Chem.

[B26] Chen L, He C, Baoprasertkul P, Xu P, Li P, Serapion J, Waldbieser G, Wolters W, Liu Z (2005). Analysis of a catfish gene resembling interleukin-8: cDNA cloning, gene structure, and expression after infection with Edwardsiella ictaluri. Dev Comp Immunol.

[B27] Fernandez PC, Frank SR, Wang L, Schroeder M, Liu S, Greene J, Cocito A, Amati B (2003). Genomic targets of the human c-Myc protein. Genes Dev.

[B28] Geiser T, Dewald B, Ehrengruber MU, Clark-Lewis I, Baggiolini M (1993). The interleukin-8-related chemotactic cytokines GRO alpha, GRO beta, and GRO gamma activate human neutrophil and basophil leukocytes. J Biol Chem.

[B29] Wiens GD, Glenney GW, Lapatra SE, Welch TJ (2006). Identification of novel rainbow trout (Onchorynchus mykiss) chemokines, CXCd1 and CXCd2: mRNA expression after Yersinia ruckeri vaccination and challenge. Immunogenetics.

[B30] Lee J, Cacalano G, Camerato T, Toy K, Moore MW, Wood WI (1995). Chemokine binding and activities mediated by the mouse IL-8 receptor. J Immunol.

[B31] Frohman MA (1993). Rapid amplification of complementary DNA ends for generation of full-length complementary DNAs: thermal RACE. Methods Enzymol.

[B32] Altschul SF, Gish W, Miller W, Myers EW, Lipman DJ (1990). Basic local alignment search tool. J Mol Biol.

[B33] Thompson JD, Higgins DG, Gibson TJ (1994). CLUSTAL W: improving the sensitivity of progressive multiple sequence alignment through sequence weighting, position-specific gap penalties and weight matrix choice. Nucleic Acids Res.

[B34] Nielsen H, Engelbrecht J, Brunak S, von Heijne G (1997). Identification of prokaryotic and eukaryotic signal peptides and prediction of their cleavage sites. Protein Eng.

[B35] Apweiler R, Attwood TK, Bairoch A, Bateman A, Birney E, Biswas M, Bucher P, Cerutti L, Corpet F, Croning MD (2001). The InterPro database, an integrated documentation resource for protein families, domains and functional sites. Nucleic Acids Res.

[B36] Schwede T, Kopp J, Guex N, Peitsch MC (2003). SWISS-MODEL: An automated protein homology-modeling server. Nucleic Acids Res.

[B37] Guex N, Peitsch MC (1997). SWISS-MODEL and the Swiss-PdbViewer: an environment for comparative protein modeling. Electrophoresis.

[B38] Kumar S, Tamura K, Nei M (2004). MEGA3: Integrated software for Molecular Evolutionary Genetics Analysis and sequence alignment. Brief Bioinform.

[B39] Solem STJJB, Robertsen B (1995). Stimulation of respiratory burst and phagocytic activity in Atlantic salmon (Salmo salar L.) macrophages by lipopolysaccharide. Fish Shellfish Immunol.

[B40] Falk W, Goodwin RH, Leonard EJ (1980). A 48-well micro chemotaxis assembly for rapid and accurate measurement of leukocyte migration. J Immunol Methods.

