# Supplementary material for: Cloning and bioactivity analysis of a CXC ligand in black seabream Acanthopagrus schlegeli: the evolutionary clues of ELR+CXC chemokines
Source: BMC Immunol. 2008 Nov 7;9:66. doi: 10.1186/1471-2172-9-66 (PMC2585555; doi:10.1186/1471-2172-9-66)
Supplement: Additional file 1 — Table for cxc primer [file 1471-2172-9-66-S1.doc]

**Table 1. The sequence of primers**

| dCXC-F | 5′ -TGC CRC TGC ATH GAR AC-3′ |
| --- | --- |
| dCXC-R | 5′ -ACT TGT TVA TGA CYH TCT TVA CCC A -3′  R=G, A; H=A, T, C; V=A, C, G; Y=T, C |
| adapter dT | 5’-GGC CAC GCG TCG ACT AGT ACT_17_-3’ |
| oligo dT | 5-TTT TGT ACA AGC T_17_ N1N -3´  (N1=A, C, G; N=A, T, C, G) |
| F85 | 5'-GAG ACC GAG ATC ATT GCC ACT CTG A-3' |
| adapter | 5’-GGC CAC GCG TCG ACT AGT AC-3’ |
| R94 | 5'-GGC AGG AAT CAG CTC CAC CTT CA- 3' |
| Oligo dG | 5’-GGC CAC GCG TCG ACT AGT ACG_15_-3’ |
| GR | 5'-CAG ATT GTC AGA GCC AGA-3' |
| b-actinF | 5'-A TCG TGG GGC GCC CCA GGC ACC-3' |
| b-actinR | 5'-CTC CTT AAT GTC ACG CAC GAT TTC-3' |
| rCXC F | 5'-G TGG ATC CAG CCT GGG AGT GGA-3' |
| rCXC R | 5'-CAA AGC TTT CAA CGT CTC GCG TTG-3' |
| pQE30-R | 5’-G TTC TGA GGT CAT TAC TGG-3’ |
| pQE30-F | 5’-CCC GAA AAG TGC CAC CTG-3’ |
